# Catabolism of fats and branched‐chain amino acids in children with Type 1 diabetes: Association with glycaemic control and total daily insulin dose

**DOI:** 10.1002/edm2.448

**Published:** 2023-09-16

**Authors:** Grace Hendrix, Yuliya Lokhnygina, Megan Ramaker, Olga Ilkayeva, Michael Muehlbauer, William Evans, Lisa Rasbach, Robert Benjamin, Michael Freemark, Pinar Gumus Balikcioglu

**Affiliations:** ^1^ Division of Pediatric Endocrinology and Diabetes Duke University Medical Center Durham North Carolina USA; ^2^ Department of Biostatistics and Bioinformatics Duke University Durham North Carolina USA; ^3^ Duke Molecular Physiology Institute and Sarah W. Stedman Nutrition and Metabolism Center Duke University Medical Center Durham North Carolina USA; ^4^ Department of Medicine, Division of Endocrinology, Metabolism, and Nutrition Duke University School of Medicine Durham North Carolina USA; ^5^ University of California‐Berkeley Berkeley California USA; ^6^ Duke University Durham North Carolina USA

**Keywords:** branched‐chain amino acid, fatty acid oxidation, insulin resistance, metabolomics, Type 1 diabetes

## Abstract

**Objective:**

Hyperglycaemia in Type 1 diabetes (T1D) results from an absolute insulin deficiency. However, insulin resistance (IR) may exacerbate glycaemic instability in T1D and contribute to long‐term cardiovascular complications. We previously showed that IR in teenagers with obesity is associated with sex‐dependent derangements in the catabolism of branched‐chain amino acids (BCAA) and fatty acids. Here we hypothesized that byproducts of BCAA and fatty acid metabolism may serve as biomarkers or determinants of glycaemic control and IR in prepubertal or early pubertal children with T1D.

**Methods:**

Metabolites, hormones and cytokines from fasting blood samples were analysed in 28 children (15 females, 13 males; age 6–11 years) with T1D. Principal components analysis (PCA) and multiple linear regression models were used to correlate metabolites of interest with glycaemic control, total daily insulin dose (TDD, units/kg/d), adiponectin and the triglyceride (TG) to high‐density lipoprotein (HDL) ratio.

**Results:**

Males and females were comparable in age, BMI‐z, insulin sensitivity, glycaemic control, inflammatory markers, BCAAs and C2/C3/C5‐acylcarnitines. The majority of components retained in PCA were related to fatty acid oxidation (FAO) and BCAA catabolism. HbA1c correlated positively with Factor 2 (acylcarnitines, incomplete FAO) and Factor 9 (fasting glucose). TDD correlated negatively with C3 and C5 and Factor 10 (BCAA catabolism) and positively with the ratio of C2 to C3 + C5 and Factor 9 (fasting glucose).

**Conclusions:**

These findings suggest that glucose intolerance in prepubertal or early pubertal children with T1D is accompanied by incomplete FAO while TDD is associated with preferential catabolism of fats relative to amino acids.

## Graphical abstract

We hypothesized that byproducts of BCAA and fatty acid metabolism may serve as biomarkers or determinants of glycaemic control and insulin resistance (IR) in prepubertal or early pubertal children with Type 1 diabetes (T1D). Principal components analysis and multiple linear regression models were used to correlate metabolites of interest with glycaemic control and IR. Our findings suggest that glucose intolerance in prepubertal or early pubertal children with T1D is accompanied by incomplete fatty acid oxidation while total daily insulin dose is associated with preferential catabolism of fats relative to amino acids.

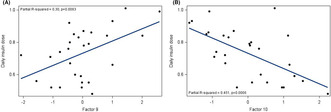

## 1 INTRODUCTION

Hyperglycaemia in Type 1 diabetes (T1D) results from an absolute deficiency of insulin. However, resistance to insulin action may exacerbate glycaemic instability in T1D and contribute to long‐term cardiovascular complications.^1^ Insulin sensitivity in adolescents with T1D has been shown to be inversely associated with cardiovascular disease risk factors including elevated blood pressure, fasting total and low‐density lipoprotein cholesterol (LDL), high sensitivity C‐reactive protein (hs‐CRP) and body mass index (BMI) z‐score.^1^ Further investigation of the association between insulin resistance (IR) and T1D with the development of therapeutic measures directed at improving insulin sensitivity may help to improve outcomes for youth with T1D.

Mechanisms explaining the pathogenesis of IR in T1D remain poorly understood but likely involve dysregulation of insulin action in liver, skeletal muscle and adipose tissues. This in turn promotes lipolysis and proteolysis and decreases glucose uptake and utilisation.^2^, ^3^ Factors postulated to contribute to IR in T1D include free fatty acidaemia,^2^ growth hormone (GH) hypersecretion,^4^, ^5^, ^6^, ^7^, ^8^ hypercortisolaemia,^4^, ^9^, ^10^ excess adiposity^11^ and pubertal progression, especially in girls.^12^, ^13^ While the prevalence of obesity is increasing in individuals with T1D, studies have also demonstrated that normal‐weight adolescents and adults with T1D are less sensitive to insulin than controls without T1D with similar BMI.^3^, ^14^, ^15^, ^16^

We previously showed that IR in adolescents with obesity is associated with sex‐dependent derangements in the catabolism of branched‐chain amino acids (BCAA) and fatty acids.^17^ Here we hypothesized that byproducts of BCAA and fatty acid catabolism may serve as biomarkers or determinants of glycaemic control and IR in prepubertal and early pubertal children with T1D. To test that hypothesis, we used metabolomic profiling, principal components analysis (PCA) and multiple linear regression models to assess the correlations between amino acids and fatty acid metabolites and surrogate markers of glycaemic control and IR. Glycaemic control was evaluated by haemoglobin A1c (HbA1c), fasting blood glucose and time in range (TIR) from continuous glucose monitoring (CGM) downloads. Surrogate measures of IR included total and high molecular weight adiponectin, and the triglyceride (TG) to high‐density lipoprotein (HDL) ratio. We also measured total daily insulin dose (TDD, units/kg/d), which can reflect in part the sensitivity to insulin action^12^, ^13^ as well as the daily macronutrient intake and energy expenditure. In poorly controlled individuals with T1D, hypercortisolaemia and elevated cytokines can induce proteolysis and muscle catabolism and reduce muscle protein synthesis.^18^, ^19^ This is proposed to cause a reduction in muscle mass, which may limit insulin‐dependent glucose uptake and thereby exacerbate glucose intolerance. We therefore measured fasting cortisol and inflammatory cytokines. Finally, leptin was included as a surrogate measure of adipose tissue mass.

We focused on young prepubertal and early pubertal children with T1D to minimize the effects of sex steroids and pubertal increases in GH on glycaemic control and insulin sensitivity. Our aims were to, (1) identify potential biomarkers that associate with glucose intolerance and IR in children with T1D; and (2) determine if there are any sex differences in these biomarkers in prepubertal and early pubertal children with T1D.

## 2 RESEARCH DESIGN AND METHODS

### 2.1 Participants

Peripheral sensitivity to insulin declines during puberty, reaching a nadir at Tanner Stage III.^20^, ^21^, ^22^ We limited our cohort to young (age ≥6–11 years), prepubertal and early pubertal children to minimize pubertal effects on insulin sensitivity. Participants with T1D were recruited from the Duke University Pediatric Type 1 Diabetes Clinics. All children had positive glutamic acid decarboxylase, islet cell and/or insulin autoantibodies and were insulin dependent. Inclusion criteria included diagnosis of T1D for at least 3 months, glycaemic stability (defined by no metabolic decompensation at the time of recruitment or study‐related activities) and weight maintenance (no recent significant weight loss or gain). Subjects were excluded if they had current or recent (within the past month) use of medications affecting insulin sensitivity (oral or inhaled steroids, metformin, thiazolidinediones or atypical antipsychotics); carried a diagnosis of a genetic syndrome causing diabetes; had untreated hypothyroidism; or had anaemia or haemoglobinopathies that could affect HbA1c levels. The protocol was approved by Duke University's Institutional Review Board. The parent/guardian provided written consent; and the participant provided written assent as appropriate for age.

Twenty‐nine participants were enrolled; one patient withdrew from the study; thus, the study cohort consisted of 28 prepubertal or early pubertal youth with T1D (15 females and 13 males).

### 2.2 Anthropometric measurements

Body weight, measured to the nearest 0.1 kg, and height, measured to the nearest 0.1 cm, were measured by standard methods. BMI, BMI percentiles and BMI z‐scores were calculated using the SAS program available at Centers for Disease Control and Prevention (https://www.cdc.gov/nccdphp/dnpao/growthcharts/resources/sas.htm). Body fat percentage (BF%) was estimated by electrical impedance using a Tanita BC‐418 segmental body composition analyzer. A physical examination was performed by a paediatric endocrinologist or a nurse practitioner specialized in paediatric endocrinology and diabetes.

### 2.3 Laboratory analysis

Annual screening bloodwork including thyroid profile, lipid panel and celiac disease screening, as well as, HbA1c were extracted from medical charts. After an 8–12 hour fast, a fasting plasma sample (5 mL whole blood) was collected to assess surrogate measures of insulin sensitivity, conventional metabolites, plasma acylcarnitines, amino acids, cortisol and inflammatory markers. All samples were stored until analysis was run together in a batch.

#### 2.3.1 Adiponectin and leptin

Total and high molecular weight (HMW) adiponectin were measured using ELISA kits from Alpco (Salem, NH) for which plasma samples were diluted 1000‐fold. Leptin was measured using immunoassay kits from Meso Scale Discovery (MSD) on an SI‐2400 electrochemiluminescent imager. Reproducibility, as assessed by coefficient of variation for duplicate measurements, averaged 5.6%.

#### 2.3.2 Conventional metabolite analysis

Measurements of conventional metabolites were performed using a Beckman‐Coulter DxC 600 clinical analyzer (Brea, CA). Plasma glucose, total cholesterol, HDL, LDL and triglycerides (TGs) were measured using reagents from Beckman. Total nonesterified fatty acids (NEFA), total ketones and 3‐hydroxybutyrate were measured with reagents from Wako (Osaka, Japan).

#### 2.3.3 Plasma acylcarnitines and amino acids

Acylcarnitines (0.01–40 μmol/L, 15%) and amino acids (5–1000 μmol/L, 15%) were analysed by tandem mass spectrometry using a Waters TQD instrument.^23^, ^24^

#### 2.3.4 Inflammatory markers

Cytokines, including interferon gamma (IFN‐γ), interleukin 6 (IL‐6), interleukin 10 (IL‐10) and tumour necrosis factor alpha (TNF‐α), were measured by multiplex assay using the MSD imager. High‐sensitivity CRP (hs‐CRP) was measured using the analyser and reagents from Beckman. Cortisol was assayed using a kit from Alpco (Salem, NH).

#### 2.3.5 Glycaemic control

Fasting plasma glucose, HbA1c and insulin regimens were extracted from medical charts. Of our 28 patients, 11 were on multiple daily injections (MDI) and 17 were using insulin pumps (9 on Omnipod, 7 on Tslim and 1 on Medtronic). When available, continuous glucose monitoring (CGM) data were also extracted from medical charts, which provided time in range (TIR). All 28 patients were using CGM systems. We included 2 weeks of CGM data from the weeks preceding the study visit to reflect current metabolic status and accepted readouts reflecting >70% CGM use per established protocol.^25^ During this 2‐week period, the mean percentage of days with CGM data was 89.3 ± 17.9 [range of 27%–100% with only 4 patients falling below 70%] and the mean time during which the CGM was active was 91.2 ± 13.1 [range of 45.4%–99.4% with only 4 patients falling below 90%].

### 2.4 Statistical analysis

Sample size was calculated using SAS (SAS Institute, Inc.) Proc Power to detect correlations of 0.5 or greater between metabolites of interest and TDD. In linear regression model with five explanatory variables, a sample size of 27 provides power of 80% to detect a correlation of 0.5 using a two‐sided 0.05 significance level test; thus, our sample size (*n* = 28) provides adequate statistical power for our primary analysis of multivariable models of IR. Between group comparisons of the participant characteristics, participant body composition measures and measures of glycaemic control/insulin sensitivity were performed by *t*‐test if the variable was normally distributed or by Wilcoxon rank sum test when not normally distributed. We calculated Spearman correlation coefficients to analyse bivariate relationships between surrogate measures of IR, and BCAA and FAO metabolites. All analyses were performed in R version 4.1.2 (2021‐11‐01) and SAS version 9.4 (SAS Institute, Inc.). Descriptive results in tables are reported as mean ± SD. *p*‐value ≤ .05 was considered statistically significant.

### 2.5 Principal components analysis

Principal components analysis (PCA) with varimax rotation was used to reduce the large number of correlated metabolites into uncorrelated clusters of fewer components.^26^, ^27^ These factors were then used as explanatory variables in multivariable models of IR.

All metabolites were assessed for normality and those that were not normally distributed were log‐transformed. Eleven principal components with the largest eigenvalues that explained 82% of the variance of all metabolites used for the analysis were retained for downstream analysis. Metabolites with an absolute value of factor loading of 0.4 or greater were considered to constitute that component.

For modelling, the components retained in the PCA were used in multivariable models as explanatory variables. None of the dependent variables required transformation to improve the normality assumption. Model selection was performed using stepwise selection with both entry and stay criteria set to *p* = .05. All models were adjusted for age, sex, Tanner stage and diabetes duration.

## 3 RESULTS

### 3.1 Participant characteristics and anthropometric measurements

Twenty‐eight subjects were studied; 15 females and 13 males. This included 82.1% (23 out of 28) Caucasian/White, 10.7% (3 out of 28) Black/African American, 3.6% (1 out of 28) Native Hawaiian or Other Pacific Islander and 3.6% (1 out of 28) two or more races. Ethnicity was reported as 89.3% (25 out of 28) non‐Hispanic/Latino, 7.1% (2 out of 28) Hispanic/Latino and 3.6% (1 out of 28) not reported. Mean diabetes duration was 49.4 ± 30.7 months.

Male participants had mean weight 33.75 ± 6.17 kg, mean height 137.55 ± 7.96 cm, mean BMI z‐score 0.367 ± 0.98 and mean BF% 19.93 ± 5.41 (Table 1). Female participants had mean weight of 36.57 ± 7.07 kg, mean height 139.23 ± 7.66 cm, mean BMI z‐score 0.680 ± 0.85 and mean BF% 24.67 ± 7.04 (Table 1). Anthropometric measures, body composition and pubertal stage was comparable between female and male participants (Table 1).

**TABLE 1:** Comparisons of anthropometric values, insulin sensitivity measures, metabolites, glycaemic control and inflammatory markers among males and females.

	Males	Females	*p* Value
	*n* = 13	*n* = 15	
Anthropometric values			
Age, years	9.31 ± 1.49	9.00 ± 1.31	.570
Weight (kg)	33.75 ± 6.17	36.57 ± 7.07	.270
Height (cm)	137.55 ± 7.96	139.23 ± 7.66	.576
BMI Z‐score	0.367 ± 0.98	0.680 ± 0.85	.379
BF (%)	19.93 ± 5.41	24.67 ± 7.04	.109
Insulin sensitivity measures			
Total adiponectin (ng/mL)	8288.08 ± 3203.14	8898.67 ± 2163.72	.496
HMW adiponectin (ng/mL)	3826.15 ± 2144.34	4373.00 ± 1510.88	.274
Daily insulin requirement (IU/kg/day)	0.70 ± 0.14	0.76 ± 0.17	.300
Leptin (pg/mL)	5237.92 ± 4376.98	6661.20 ± 5822.74	.413
TG (mg/dL)	39.21 ± 8.57	49.39 ± 13.55	.028
HDL (mg/dL)	63.54 ± 9.97	66.72 ± 6.50	.320
TG/HDL ratio	0.62 ± 0.11	0.74 ± 0.20	.059
Metabolites			
Glucose (mg/dL)	171.23 ± 49.51	171.4 ± 51.85	.945
Total ketones (umol/L)	323.75 ± 262.34	324.41 ± 262.34	1.000
NEFA (mmol/L)	0.71 ± 0.32	0.59 ± 0.22	.279
Glutamate (μM)	84.89 ± 17.60	83.95 ± 17.45	.964
Valine (μM)	219.25 ± 18.72	225.29 ± 33.92	.683
Leucine/isoleucine (μM)	172.53 ± 22.84	165.93 ± 22.85	.467
BCAA (μM)	391.78 ± 35.46	391.22 ± 50.25	.856
C3 (μM)	0.23 ± 0.06	0.22 ± 0.07	.786
C5 (μM)	0.10 ± 0.03	0.10 ± 0.03	1.000
C2 (μM)	8.04 ± 2.18	7.64 ± 2.46	.751
C2/C3 + C5	26.13 ± 9.63	24.29 ± 8.75	.496
Glycaemic control parameters			
Time in range (%)	57.12 ± 13.06	55.00 ± 7.85	.720
HbA1c (%)	7.15 ± 1.03	7.69 ± 1.22	.212
Inflammatory markers			
IFN‐γ (pg/mL)	14.06 ± 4.61	31.99 ± 55.29	.279
IL‐6 (pg/mL)	0.54 ± 0.41	4.81 ± 16.37	.344
IL‐10 (pg/mL)	0.24 ± 0.22	0.22 ± 0.23	.945
TNFα (pg/mL)	1.79 ± 0.37	1.98 ± 0.51	.369
CRP (mg/ L)	0.32 ± 0.40	0.83 ± 0.94	.120
Cortisol (ug/dL)	10.29 ± 5.06	8.32 ± 2.78	.413

### 3.2 Metrics of glycaemic control and insulin sensitivity

HbA1c (males: 7.15 ± 1.03%; females: 7.69 ± 1.22%) and TIR (males: 57.12 ± 13.06%; females: 55.00 ± 7.85%) were comparable in males and females (Table 1). Pubertal status was positively correlated with HbA1c (*r* = .442, *p* = .0210), but not with TIR (*r* = −.269, *p* = .1741).

Total adiponectin (males: 8288.08 ± 3203.14 ng/mL; females: 8898.67 ± 2163.72 ng/mL), HMW adiponectin (males: 3826.15 ± 2144.34 ng/mL; females: 4373.00 ± 1510.88 ng/mL), TG/HDL ratio (males: 0.62 ± 0.11; females: 0.74 ± 0.20) and TDD (males: 0.70 ± 0.14 IU/kg/day; females: 0.76 ± 0.17 IU/kg/day) were comparable in males and females (Table 1). Pubertal status did not correlate with total adiponectin (*r* = .184, *p* = .36), HMW adiponectin (*r* = .245, *p* = .22), TG/HDL ratio (*r* = .171, *p* = .39) or TDD (*r* = .190, *p* = .34). BF% did not correlate with HbA1c (*r* = .244, *p* = .30), TIR (*r* = .013, *p* = .96), total adiponectin (*r* = .243, *p* = .30), HMW adiponectin (*r* = .154, *p* = .52), TG/HDL ratio (*r* = .290, *p* = .21) or TDD (*r* = −.408, *p* = .07).

### 3.3 Annual screening laboratory studies

All 28 patients had normal thyroid levels and normal (negative) celiac screening obtained per standard of care clinic visits.

### 3.4 Cortisol, inflammatory markers and leptin

There were no significant differences between males and females with regard to morning cortisol levels, cytokines including IFN‐γ, IL‐6, IL‐10, TNF‐α or hs‐CRP (Table 1). hs‐CRP and leptin levels correlated with BF% (*r* = .62, *p* = .0038 and *r* = .72, *p* = .0004, respectively).

### 3.5 Conventional metabolites

There were no significant differences in fasting glucose, NEFA, total ketones or lipids between males and females (Table 1).

### 3.6 Bivariate associations between BCAA and related byproducts and FAO metabolites, glycaemic control and surrogate measures of insulin sensitivity

C3 and C5 acylcarnitines are byproducts of BCAA catabolism. C2 acylcarnitine is an end product of complete FAO. Thus the ratio of C2/(C3 + C5) was calculated as a surrogate measure of the catabolism of fats relative to the catabolism of BCAAs. Levels of BCAAs, C2, C3 and C5, and the ratio C2/(C3 + C5) were similar among males and females (Table 1).

### 3.7 Correlation with HbA1c and TIR

TDD (daily insulin requirement, units/kg/d) correlated negatively with metrics of BCAA catabolism including C3 and C5 acylcarnitines (*r* = −.36, *p* = .05 and *r* = −.43, *p* = .02, respectively). In contrast, TDD correlated positively with a metric of fat catabolism (ratio of C2 to C3 + C5, *r* = .46, *p* = .01). There were no significant correlations between HbA1c (C3: *r* = −.032, *p* = .87; C5: *r* = .079, *p* = .69; C2/C3 + C5: *r* = .022, *p* = .91), TIR (C3: *r* = −.060, *p* = .76; C5: *r* = −.219, *p* = .26; C2/C3 + C5: *r* = .066, *p* = .74), leptin (C3: *r* = −.009, *p* = .96; C5: *r* = .145, *p* = .46; C2/C3 + C5: *r* = −.026, *p* = .89) or BF% (C3: *r* = .213, *p* = .37; C5: *r* = .102, *p* = .67; C2/C3 + C5: *r* = −.337, *p* = .15) and metrics of BCAA catabolism or fat catabolism.

### 3.8 PCA and multiple linear regression models

PCA was used to consolidate the metabolites into 11 components. These 11 principal components explained 82% of the variance of all metabolites used for the analysis. The constituents for each component are shown in Table 2. The metabolites that compose a factor were biologically related, providing a biological description for each factor. Of interest, the majority of components retained in the PCA were related to FAO and BCAA catabolism. Factor 1 was related to complete FAO (evident by C2, total ketones and 3‐HB) while Factor 2 was related to incomplete FAO (evident by short‐, medium‐ and long‐chain aclycarnitines); these explained 21% and 11% of the variance, respectively. BCAA catabolism was reflected in Factor 5 (as evidenced by urea cycle intermediates), Factor 6 (as evidenced by BCAA and C4‐DC/Ci4‐DC(−)) and Factor 10 (as evidenced by C5, BCAA byproducts).

**TABLE 2:** Factors obtained from PCA.

Factor	Metabolites within factor	Description	Eigenvalue	Proportion of variance explained	Cumulative proportion of variance explained
1	C14:1 C16:1 C16:2 C14 C14:2 C12 C2 C16:1‐OH/C14:1‐DC C16 C14:1‐OH C18:1‐OH/C16:1‐DC C12‐OH/C10‐DC C18:1 C10‐OH/C8‐DC C12:1 C14‐OH/C12‐DC C4‐OH C6 C18 C18:2 3HB C8 TK C10 C5‐OH/C3‐DC C18‐OH/C16‐DC	FAO byproducts	16.97	0.21	0.21
2	C10 C8 C10:1 C18:1‐DC C4‐Ci4 C6 C7‐DC C22 C14‐OH/C12‐DC C10‐OH/C8‐DC C12‐OH/C10‐DC C20‐OH/C18‐DC	Short‐, medium‐ and long‐chain acylcarnitines	7.53	0.11	0.32
3	C10:3 C10:2 C8:1 C8:1‐DC C6‐DC/C8‐OH C12:1 C8:1‐OH/C6:1‐DC NEFA(−) 3HB(−) His(−) C10:1 TK(−)	FAO byproducts	5.66	0.09	0.41
4	Ser Glx Orn Gly C8:1‐OH/C6:1‐DC(−) NEFA C18:2	AA and NEFA	4.18	0.07	0.48
5	Arg Cit CRP(−) Met C18:2(−)	Urea cycle intermediates and CRP	3.75	0.06	0.54
6	Phe Leu:Ile Met Tyr Val His Orn C4‐DC/Ci4‐DC(−)	BCAA and LNAA	3.37	0.06	0.60
7	C5:1 C18 Ala 3HB(−) TK(−)	Ketones and ala	3.00	0.05	0.65
8	Asx TK 3HB C20:4(−) C5‐OH/C3‐DC	Ketones and BCAA byproducts	2.73	0.05	0.70
9	Glucose Pro His(−) CRP	Glucose	2.24	0.04	0.74
10	C18:2‐OH C5 Val	BCAA byproducts	1.87	0.04	0.78
11	C20:4 C18‐OH/C16‐DC C20 C16‐OH/C14‐DC(−)	Long‐chain acylcarnitines	1.79	0.04	0.82

**FIGURE 1:**
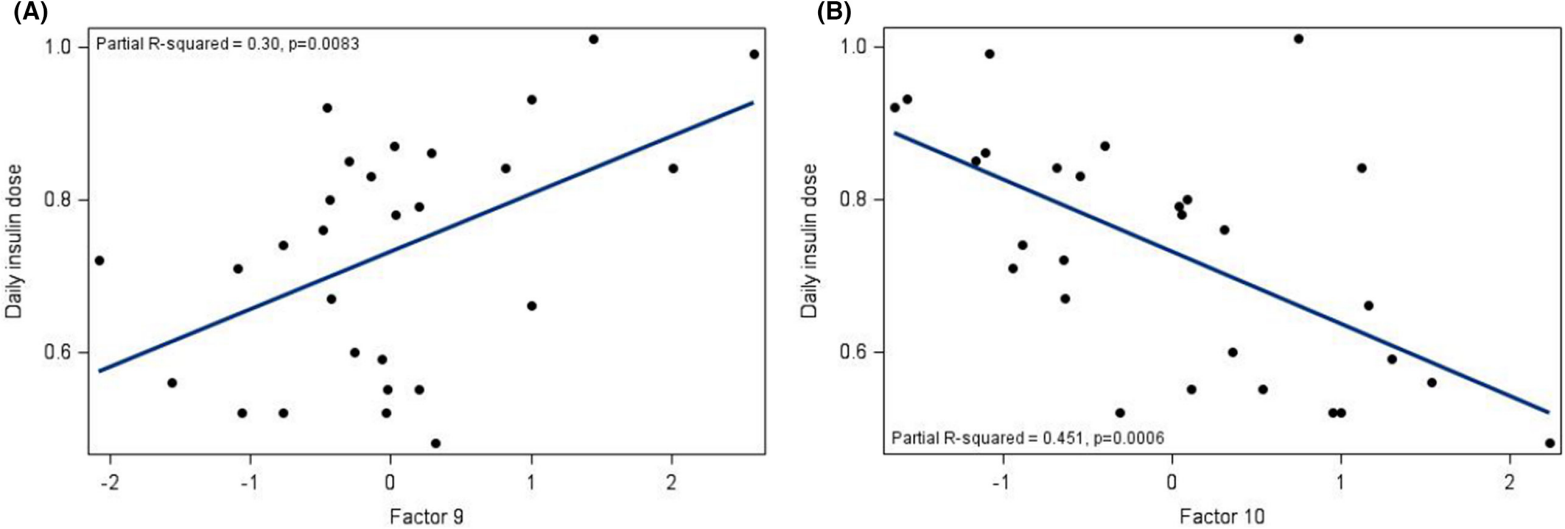
(A) Associations between total daily insulin dose (units/kg/d) and Factor 9 (glucose). (B) Associations between total daily insulin dose (units/kg/d) and Factor 10 (BCAA byproducts).

**FIGURE 2:**
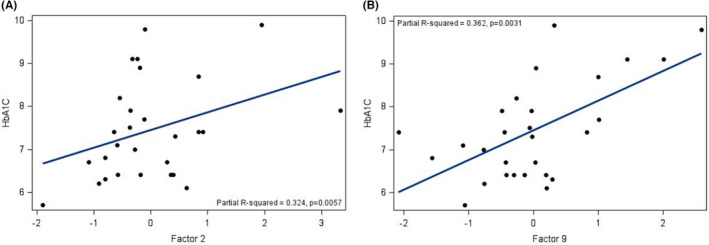
(A) Associations between HbA1c and Factor 2 (short‐, medium‐ and long‐chain acylcarnitines). (B) Associations between HbA1c and Factor 9 (glucose).

Multilinear regression models were then used to determine factors associated with glycaemic control and surrogate measures of insulin sensitivity. A summary of significant associations is shown in Table 3. HbA1c correlated positively (as expected) with Factor 9 (fasting glucose) and with Factor 2 (short‐, medium‐ and long‐chain acylcarnitines, markers of incomplete fatty acid oxidation (FAO); Table 3 and Figure 2). We did not find similar associations when looking at time in range (TIR) as our marker of glycaemic control; this may be due to the shorter time frame of 14 days used for estimating TIR, compared to HbA1c. Nevertheless, HbA1c was negatively associated with TIR (*r* = −.768; *p* < .0001), validating our data. TDD (units/kg/d) correlated positively with Factor 9 (fasting glucose), and negatively with Factor 10 (BCAA catabolism), consistent with our bivariate results (Table 3 and Figure 1). Neither total or HMW adiponectin nor TG/HDL ratio correlated significantly with any factors (Table 3).

**TABLE 3:** Summary of baseline factors associated with insulin sensitivity and glycaemic control (all models were adjusted for sex, age, Tanner stage and duration of diabetes).

Full sample	Parameter estimate	*t* Value	*p* Value
Daily insulin dose (*r* ^2^ = .60; *p* = .0029)^a^			
Female sex	0.0568	1.28	.22
Age	0.0078	0.40	.69
Tanner stage = 1 (vs. 2)	0.0081	0.13	.90
Duration of diabetes	−0.0003	−0.35	.73
Factor 9: Glucose	0.0789	2.93	.0083
Factor 10: BCAA catabolism	−0.0985	−4.05	.0006
HbA1c (*r* ^2^ = .71; *p* = .0002)			
Female sex	−0.0001	0.00	>.99
Age	−0.4243	−3.65	.0016
Tanner Stage = 1 (vs. 2)	−0.8677	−2.18	.042
Duration of diabetes	0.0088	1.76	.095
Factor 2: Acylcarnitines	0.4209	3.09	.0057
Factor 9: Glucose	0.5537	3.37	.0031

^a^ Model *R*‐squared and model *p*‐value.

## 4 DISCUSSION

Large population‐based studies have shown that patients with T1D with HbA1c levels of ≤6.9%, consistent with target control, still have increased mortality and diabetes‐related complications.^28^ Therefore, it is not possible to predict with certainty who will develop diabetic complications using current standard of care measures such as HbA1c. As insulin sensitivity in adolescents with T1D has been shown to be inversely associated with cardiovascular disease risk factors,^1^ studies focused on IR may play a key role in identifying potential biomarkers for future diabetic complications and may provide therapeutic targets.

Comprehensive metabolomic profiling has provided new insight into the mechanisms underlying IR. For example, multiple studies demonstrate increases in BCAAs in animal models of T1D,^29^, ^30^, ^31^, ^32^ adults with obesity and Type 2 diabetes^24^, ^32^, ^33^, ^34^, ^35^, ^36^, ^37^, ^38^, ^39^ and children and adolescents with obesity and IR.^17^, ^40^, ^41^, ^42^ Metabolomics has also recently been applied to predict the development or progression to T1D in children^43^, ^44^, ^45^, ^46^, ^47^, ^48^ but studies of children with active T1D are limited. A recent investigation of urine samples of children with T1D and non‐diabetic controls did not assess the relationship between metabolite levels and insulin action or glucose tolerance.^10^ This study found increased urinary cortisol and its metabolites in children with T1D, when compared with healthy controls; however, these metabolites did not correlate with measures of glycaemic control (HbA1c), insulin sensitivity or pubertal status.^10^ Our study used metabolomic profiling, principal components analysis and multiple linear regression models to assess the correlations between BCAA and fatty acid metabolites and glycaemic control and surrogate markers of IR in young, prepubertal or early pubertal children with T1D.

Our findings include three novel observations. First, glycaemic control, as assessed by HbA1c, correlated with metabolites of incomplete FAO but not with ketones or acetylcarnitine, the markers of complete FAO. Second, daily insulin requirement (TDD, units/kg/d) correlated negatively with metrics of BCAA catabolism and positively with the metrics of fat catabolism, implicating preferential catabolism of fats relative to amino acids. Finally, there were no significant differences in metabolites between males and females in our cohort of prepubertal or very early pubertal children with T1D. This observation contrasts strongly with studies of older, more sexually mature teenagers and adults, which find that fasting BCAA levels and C3 and C5 acylcarnitines are significantly higher in males than females.^17^

Surrogate metrics of IR in this study included total and high molecular weight adiponectin and the TG to HDL ratio. Adiponectin is a marker of insulin sensitivity, as it decreases hepatic glucose output and increases glucose uptake and FAO in muscles.^49^, ^50^, ^51^ The ratio of TG to HDL has previously been associated with IR in obese adolescents.^52^ Total daily insulin dose (TDD, units/kg/d) correlates inversely with whole body insulin sensitivity^12^, ^13^ but is influenced by various other factors, including patient age, macronutrient intake, energy expenditure and pubertal stage, given that increases in sex steroids and growth hormone are associated with decreased insulin sensitivity and consequent increases in insulin.^12^, ^13^ About 80% of our subjects were prepubertal, and the remaining were in early puberty. Although there was a significant positive correlation between pubertal status and HbA1c, there were no significant correlations between pubertal status and other metrics of glycaemic control (TIR), insulin sensitivity (total and HMW adiponectin, TG/HDL ratio) or TDD (units/kg/d). This is not surprising, as IR in puberty does not peak until Tanner Stage III.^20^, ^21^, ^22^

We found that daily insulin requirement (TDD, units/kg/d) correlated negatively with metrics of BCAA catabolism and positively with the ratio of acetylcarnitine (C2) to the sum of propionylcarnitine (C3) and (C5), implicating preferential catabolism of fats relative to amino acids. This is in agreement with prior studies completed in pubertal children.^53^ Additionally, our PCA and modelling demonstrated that total daily insulin dose (TDD, units/kg/d) correlated positively with Factor 9 (fasting glucose), and negatively with Factor 10 (BCAA catabolism). These findings suggest impaired catabolism of BCAA; whether impaired BCAA catabolism is a consequence or cause (or both) of IR in T1D is currently unclear. Studies have demonstrated that the changes in diabetes that can lead to cachexia and decreased muscle mass are associated with increased proteolysis and branched‐chain keto acid dehydrogenase (BCKD) activity in muscle, as well as, decreased uptake and catabolism of BCAAs.^54^ The resulting increase in BCAAs is thought to interfere with FAO in muscle, leading to an accumulation of various acylcarnitines that may contribute to IR.^54^

In contrast to daily insulin requirement, HbA1c did not associate with markers of BCAA catabolism or complete FAO. Rather, HbA1c correlated most strongly with Factor 2 (acylcarnitines, markers of incomplete FAO) and Factor 9 (glucose). The association of glycaemic control in T1D with fatty acid metabolism has been noted previously: hyperinsulinaemic euglycaemic clamp studies demonstrated that adolescents with T1D have significantly higher rates of lipolysis and endogenous glucose production and lower peripheral glucose uptake during hyperinsulinaemia compared to controls.^55^ Higher rates of lipolysis, free fatty acidaemia^2^ and glucose production could reflect combined effects of insulin deficiency, growth hormone hypersecretion^4^, ^5^, ^6^, ^7^, ^8^ and/or hypercortisolaemia,^4^, ^9^, ^10^ which are most apparent at times of severe metabolic decompensation.^56^ Increases in availability of fatty acids may lead to mitochondrial overload and stress,^32^, ^57^, ^58^, ^59^ with consequent incomplete FAO^32^, ^57^, ^60^ and decreased glucose oxidation. We did not find similar associations when looking at time in range (TIR) as our marker of glycaemic control; this may be due to the shorter time frame of 14 days used for estimating TIR, compared to HbA1c. Nevertheless, HbA1c was negatively associated with TIR (*r* = −.768; *p* < .0001), validating our data.

As noted previously, there were no significant differences in metabolites between the young males and females in our cohort with T1D. This finding contrasts sharply with our previous studies of IR in adolescents with obesity: fasting levels of BCAA and products of BCAA catabolism were significantly higher in obese teenage boys than obese girls of similar age and BMI z‐score.^17^ It is likely that metabolic changes operative during sexual maturation explain the differences between the studies. Genetically engineered mouse models demonstrate that oestrogen increases insulin sensitivity and limits upper body fat deposition, while progesterone opposes these effects.^61^, ^62^ Additionally, during puberty, total body fat content and subcutaneous fat deposition increase in girls, while body fat percentage declines and lean body mass increases in boys.^63^ Increases in body fat and reductions in lean mass are known to be associated with IR in adolescents and adults. However, other studies found no relationship between sex steroid levels and measures of carbohydrate metabolism^64^ or insulin sensitivity^65^ during puberty. Although no differences between males and females were detected in our study, the BCAA levels in our patients were elevated compared to both lean and obese controls of older children in a prior study^66^ and relatively comparable to levels found in older obese teenagers.^17^

Elevated cortisol and inflammatory cytokines have previously been demonstrated in individuals with T1D in ketoacidosis and can induce proteolysis and muscle catabolism and reduce muscle protein synthesis.^25^, ^26^ In the current study of stable and otherwise healthy children with T1D, the levels of cortisol and inflammatory cytokines were normal. Moreover, there were no significant differences in cortisol levels between males and females. Given that T1D has been associated with decreased skeletal muscle mass and impaired muscle function^59^, ^67^, ^68^, ^69^, ^70^ further investigation into the relationship between skeletal muscle mass and IR is needed, as reduction in muscle is proposed to limit insulin‐dependent glucose uptake, leading to glucose intolerance.

Our study has some limitations. The sample size was small but provided adequate statistical power for our primary analysis. We used surrogate measures of insulin sensitivity; additional methods, including insulin and glucose clamps and iv and oral glucose tolerance tests, might have provided useful information regarding insulin secretion and tissue‐specific IR.^2^, ^71^

Nevertheless, our study is one of the first to examine the relationship of BCAAs and FAO metabolites to glycaemic control and metrics of insulin sensitivity in young prepubertal or early pubertal children with T1D. Our findings suggest that TDD is associated with the preferential catabolism of fats relative to amino acids, while HbA1c is associated with incomplete FAO. Future studies are needed to examine the rates of muscle protein and fat synthesis and breakdown in healthy children and children with T1D along with their relationship to insulin administration and glycaemic control, metabolic control, growth and pubertal development.

## AUTHOR CONTRIBUTIONS

**Grace Hendrix:** Conceptualization (equal); data curation (equal); formal analysis (equal); investigation (equal); methodology (equal); project administration (equal); resources (equal); software (equal); supervision (equal); validation (equal); writing – original draft (lead); writing – review and editing (equal). **Yuliya Lokhnygina:** Data curation (equal); formal analysis (equal); investigation (equal); methodology (equal); resources (equal); software (equal); writing – review and editing (equal). **Megan Ramaker:** Data curation (equal); formal analysis (equal); investigation (equal); methodology (equal); resources (equal); software (equal); writing – review and editing (equal). **Olga Ilkayeva:** Data curation (equal); formal analysis (equal); methodology (equal); resources (equal); software (equal); supervision (equal); writing – review and editing (equal). **Michael Muehlbauer:** Data curation (equal); formal analysis (equal); methodology (equal); resources (equal); software (equal); supervision (equal); writing – review and editing (equal). **William Evans:** Conceptualization (equal); investigation (equal); methodology (equal); resources (equal); validation (equal); writing – review and editing (equal). **Lisa Rasbach:** Investigation (supporting); methodology (supporting); project administration (supporting); resources (supporting); validation (supporting); writing – review and editing (supporting). **Robert Benjamin:** Investigation (supporting); methodology (supporting); project administration (supporting); resources (supporting); validation (supporting); writing – review and editing (supporting). **Michael Freemark:** Conceptualization (equal); data curation (equal); investigation (equal); methodology (equal); resources (equal); supervision (equal); validation (equal); writing – review and editing (equal). **Pinar Gumus Balikcioglu:** Conceptualization (lead); data curation (lead); formal analysis (lead); funding acquisition (lead); investigation (lead); methodology (lead); project administration (equal); resources (lead); software (lead); supervision (lead); validation (lead); visualization (lead); writing – review and editing (lead).

## FUNDING INFORMATION

P.G.B. was supported by Diabetes Research Connection (DRC), National Institute of Diabetes and Digestive and Kidney Diseases of the National Institutes of Health under the award number DK117067, Gall Family Support, Duke University Pediatric Departmental Support and Duke Strong Start Award Program. OI and metabolomics assays performed at the DMPI Metabolomics Core Laboratory were supported by National Institute of Diabetes and Digestive and Kidney Diseases of the National Institutes of Health under award number P30DK124723. The content is solely the responsibility of the authors and does not necessarily represent the official views of the National Institutes of Health.

## CONFLICT OF INTEREST STATEMENT

The authors have no conflict of interest to disclose.

## ETHICS STATEMENT

The Institutional Review Board at Duke University approved the research protocol.

## Data Availability

The data that support the findings of this study are available from the corresponding author upon reasonable request.
